# Explainable Classification of Different Coloration Stages in Cherry Fruits Using a Hybrid RF-ACO Model Based on Pomological and Cherry Fly Data

**DOI:** 10.3390/insects17080831

**Published:** 2026-08-10

**Authors:** Cebrail Barut, İnanç Özgen, Halil Bolu, Bilal Alataş, Hakan Yildirim, Ali Murat Tatar

**Affiliations:** 1 Department of Continuing Education Center, Firat University, 23000 Elazig, Türkiye; cbarut@firat.edu.tr; 2 Department of Bioengineering, Faculty of Engineering, Firat University, 23000 Elazig, Türkiye; iozgen@firat.edu.tr; 3 Department of Plant Protection, Faculty of Agriculture, Dicle University, 21000 Diyarbakır, Türkiye; 4 Department of Software Engineering, Faculty of Engineering, Firat University, 23000 Elazig, Türkiye; balatas@firat.edu.tr; 5 Department of Horticulture, Faculty of Agriculture, Malatya Turgut Özal University, 44210 Malatya, Türkiye; hakan.yildirim@ozal.edu.tr; 6 Department of Animal Science, Faculty of Agriculture, Dicle University, 21000 Diyarbakır, Türkiye; tatar@dicle.edu.tr

**Keywords:** cherry, *Rhagoletis cerasi*, Explainable Artificial Intelligence, Random Forest, Ant Colony Optimization, pomological characteristics, classification

## Abstract

The coloring process in cherry fruit affects both fruit quality and the cherry fly (*Rhagoletis cerasi*). This is critically important for host preferences of major pests such as cherry fly. In this study, a randomized classification method was used to classify five distinct coloration stages of cherry fruit based on pomological characteristics and cherry fly density data. An explainable hybrid model combining Random Forest (RF) and Ant Colony Optimization (ACO) algorithms has been developed. In this study, fruit samples of the Ziraat 900 variety were collected from four different cherry orchards in Elazığ province during five different coloration stages. Pomological characteristics such as weight, width, length, height, stem length, firmness, seed weight, soluble solids content (SSC), NaOH, and acidity were determined, and adult cherry fly densities were also recorded. In the proposed method, candidate decision rules generated by the RF algorithm were optimized using the ACO algorithm, and the most distinctive rule sets were selected. The results showed that the proposed RF-ACO model achieved a 99.48% accuracy rate and exhibited higher performance than many common machine learning methods. Feature significance analysis revealed that SSC, cherry fly density, NaOH, and acidity were the most effective parameters in the classification process. The model not only provided high accuracy thanks to the explainable IF-THEN rules it generated, but also allowed for expert interpretation of the decision-making process. The findings offer significant contributions to the development of decision support systems for determining cherry ripening periods and controlling the cherry fruit fly.

## Simple Summary

Cherry ripening and coloration changes directly influence fruit quality and attract destructive pests like the cherry fruit fly. Identifying these stages accurately helps farmers plan targeted pest control, reducing unnecessary pesticide use and crop damage. In this study, fruit samples and pest fly counts were gathered across five distinct ripening stages. A smart computer model was developed by combining two artificial intelligence techniques to classify these stages using fruit physical traits, chemical properties, and pest numbers. The model achieved over 99% accuracy, outperforming many standard methods. Key findings revealed that sugar content, pest population density, and fruit acidity were the most important factors for identifying the ripening stages. Unlike complicated AI systems that act as unreadable boxes, this method produces simple, readable rules that humans can easily interpret and verify. These results provide a practical decision tool for farmers to determine the exact harvest timing and manage cherry pests effectively.

## 1. Introduction

Fruit ripening is accompanied by a series of physical, biochemical, and physiological changes that directly influence fruit quality, harvest timing, and susceptibility to insect pests. Among these changes, fruit coloration is one of the most visible indicators of the ripening process and is closely associated with variations in SSC, acidity, firmness, and other pomological characteristics. These quality attributes not only determine the commercial value of cherries but also influence the host selection and oviposition behavior of phytophagous insects [1,2,3].

One of the most destructive pests affecting cherry production is the cherry fruit fly, *Rhagoletis cerasi* (Linnaeus, 1758) (Diptera: Tephritidae) [1]. Larval feeding causes fruit decay, premature fruit drop, and substantial economic losses, which may reach 100% when no effective control measures are implemented. Since insecticide applications are effective only during a limited period before egg laying, determining the correct timing of control is essential for successful integrated pest management (IPM). Previous studies have shown that fruit coloration, SSC, acidity, firmness, and other pomological characteristics significantly influence the host selection and oviposition behavior of *R. cerasi* [3,4,5,6]. Therefore, monitoring these fruit characteristics is important for both fruit quality assessment and pest management. Monitoring pest populations using yellow sticky traps has become an important component of IPM programs. Trap captures provide valuable information regarding seasonal population dynamics and help determine the economically optimal timing for control measures. Recent studies have combined trap data with climatic and geographical variables using machine learning algorithms, particularly Random Forest (RF), to improve population prediction and support area-wide integrated pest management (AW-IPM) strategies [7,8].

Artificial intelligence (AI) has increasingly been applied to agricultural decision-support systems. Recent studies have successfully employed machine learning and deep learning techniques to predict insect population density, identify attractant compounds, classify developmental stages, and recognize fruit fly species using imaging technologies [9,10,11,12]. These studies demonstrate that AI provides effective tools for improving pest monitoring, quarantine inspection, and precision agriculture. Among AI approaches, Explainable Artificial Intelligence (XAI) has received growing attention because agricultural experts require transparent and interpretable decision-support systems rather than black-box predictions. Rule-based classifiers are particularly attractive because they generate human-readable IF–THEN rules that explicitly explain the relationships between fruit characteristics and pest behavior. Previous studies have successfully classified cherry coloration stages [13] and cherry fly population density [14] using explainable machine learning methods, demonstrating that interpretable rules can effectively support pest management decisions. Despite these promising advances, several important limitations remain. Most previous studies have focused either on identifying a specific coloration stage [13] or on classifying pest population density [14]. Comprehensive multi-class classification that covers all five cherry coloration stages while maintaining high predictive performance and model interpretability remains limited. Furthermore, many high-performing machine learning models suffer from poor explainability, reducing user confidence and limiting their practical adoption in agricultural decision-support systems.

To address these limitations, this study proposes a hybrid Random Forest–Ant Colony Optimization (RF-ACO) framework. Random Forest is first employed to generate a diverse pool of candidate decision rules, while Ant Colony Optimization (ACO) is used to identify a compact subset of highly informative and non-redundant rules. Compared with standalone machine learning and optimization approaches, the proposed RF-ACO framework provides three major advantages:

- Compact and interpretable rule generation,
- Improved generalization through optimized rule selection,
- Enhanced computational efficiency by reducing the search space before optimization.

Consequently, the proposed framework aims to achieve high classification accuracy while producing explainable decision rules that can support agricultural experts in practical decision-making [13,14].

## 2. Materials and Methods

### 2.1. Field Sampling and Plant Material

Field studies were conducted in four commercial sweet cherry orchards growing the Ziraat 900 cultivar *Prunus avium* (L.) in Elazığ province, Türkiye: Harput 1, Harput 2, Baskil 1, and Baskil 2. Each field site comprised a 5-decare orchard:

- Harput 1 and 2: Located at coordinates 38.70° N, 39.25° E (elevation: approx. 1180 m a.s.l.) and 38.72° N, 39.28° E (elevation: approx. 1210 m a.s.l.), respectively. Trees were 12–15 years old, trained to an open-center (goblet) system, and managed under drip irrigation with standard annual winter pruning.
- Baskil 1 and 2: Located at coordinates 38.55° N, 38.82° E (elevation: approx. 980 m a.s.l.) and 38.58° N, 38.85° E (elevation: approx. 1020 m a.s.l.), respectively. Trees were 10–13 years old, drip-irrigated, and maintained under standard regional Integrated Pest Management (IPM) practices with minimal chemical intervention.

**Figure 1:**
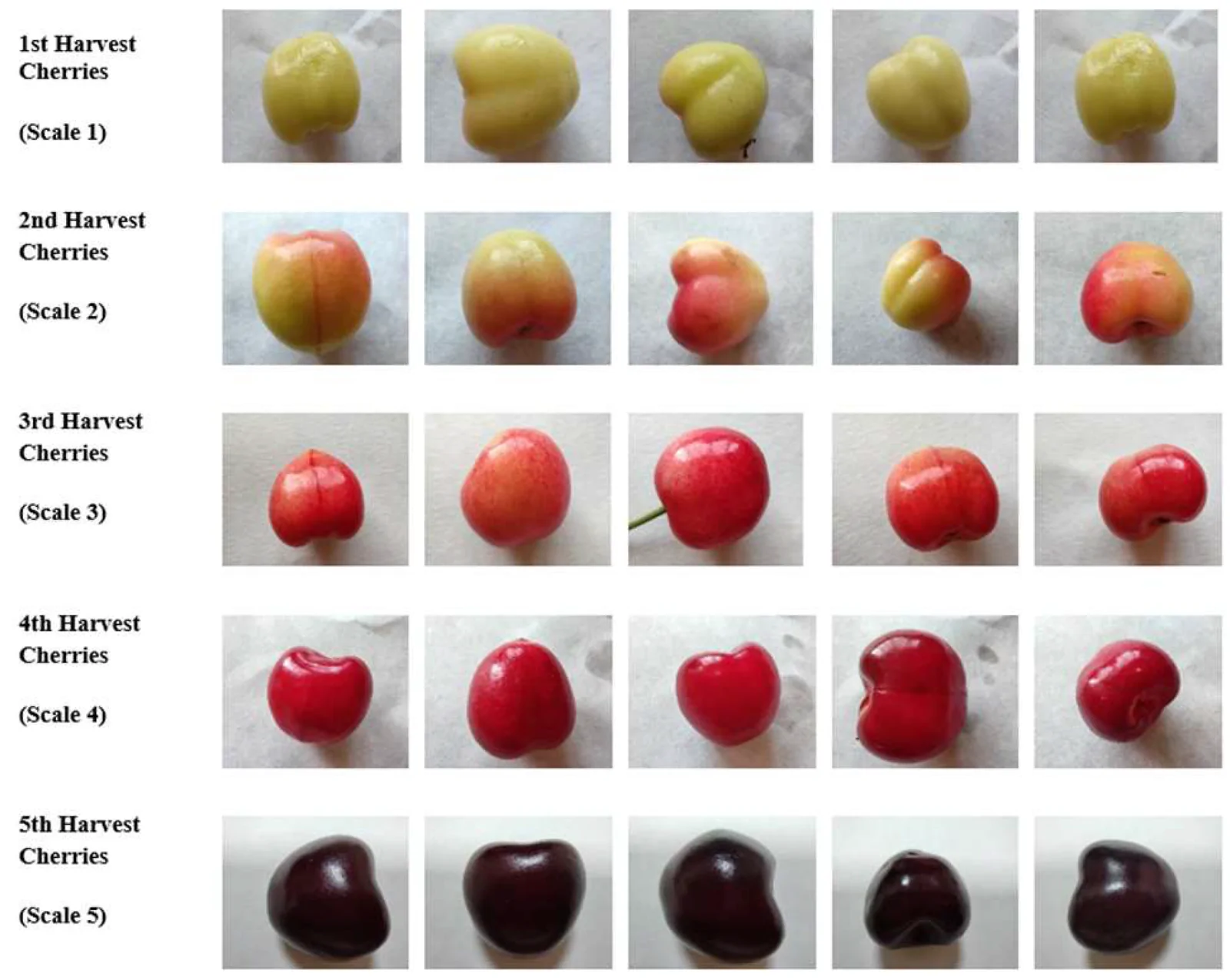
Cherry Harvesting According to Fruit Quality Characteristics.

In each orchard, adult *Rhagoletis cerasi* populations were monitored using standard yellow sticky traps (20 × 25 cm) equipped with synthetic ammonium capsule lures (*n* = 5 traps per orchard, 20 traps in total). Traps were placed from 1 April 2024 to 22 June 2024, on the outer canopy of non-border trees at a height of approximately 1.8–2.0 m facing south-southeast. Trap counts were inspected at 5- to 7-day intervals. Fruit sampling was performed from the same monitored trees across five distinct fruit coloration stages: Scale 1 (25 April 2024), Scale 2 (14 May 2024), Scale 3 (22 May 2024), Scale 4 (27 May 2024), and Scale 5 (7 June 2024) (Figure 1). A total of 381 fruit samples were systematically collected across these five ripening stages from all four locations. Collected fruit samples were immediately kept in cold storage containers (4 °C) and transferred within 12 h to the Malatya Fruit Research Institute for physical, pomological, and chemical analyses. All collected 381 fruit samples were categorized into five distinct phenological coloration target classes based on their surface pigmentation: Group A (NC: No Color/Scale 1, *n* = 71), Group B (LP: Light Pink/Scale 2, *n* = 74), Group C (MP: Medium Pink/Scale 3, *n* = 80), Group D (PR: Pinkish Red/Scale 4, *n* = 88), and Group E (FR: Full Red/Scale 5, *n* = 68).

### 2.2. Categorization and Feature Extraction of Cherry Fruit Samples

**Figure 2:**
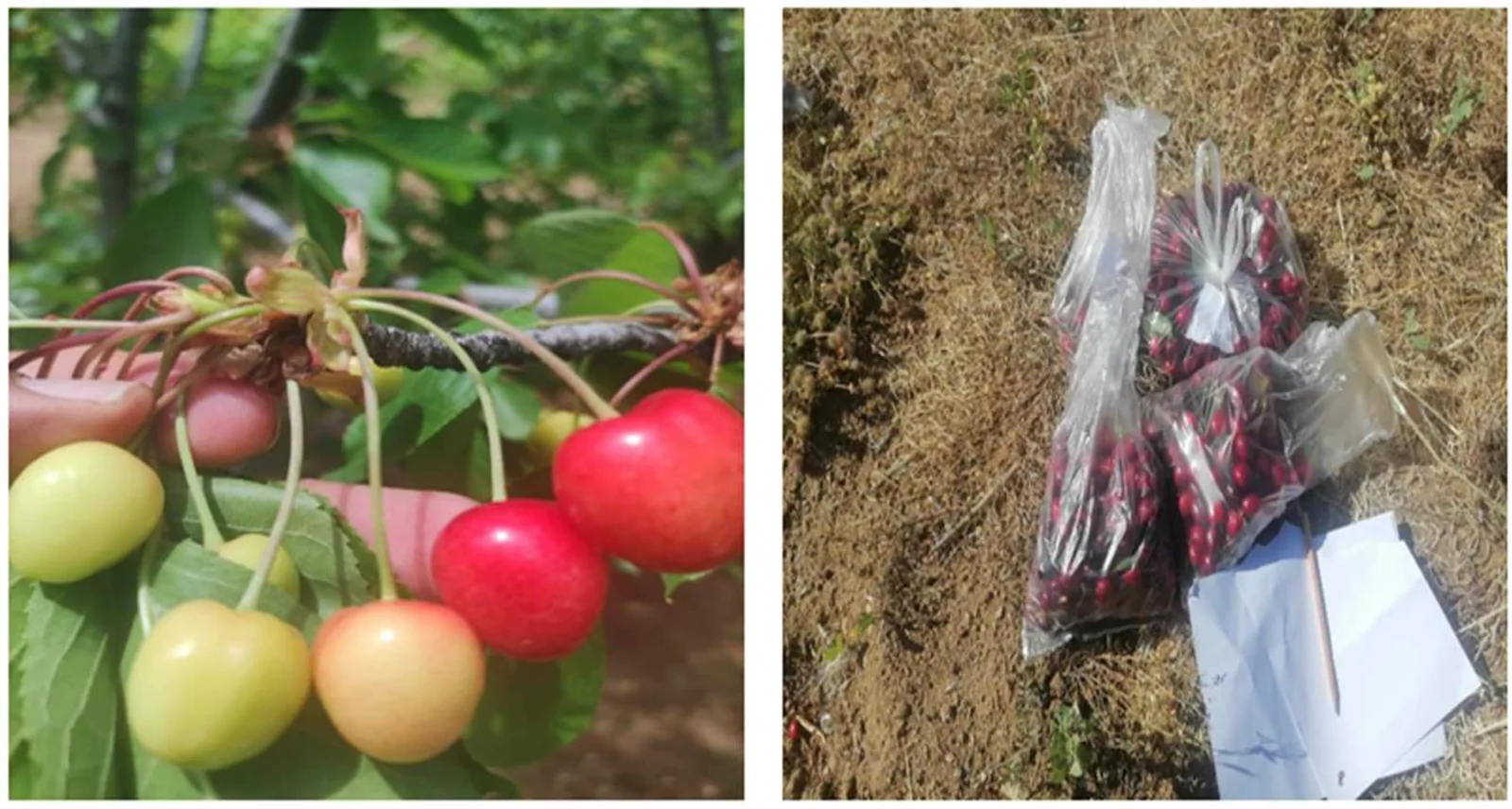
Fruit Coloration Stages Examined.

The harvested cherry fruits were classified into five distinct maturation groups based on their surface coloration profiles, as visually documented in Figure 1 and Figure 2: Group A (No color/Scale 1), Group B (Light Pink/Scale 2), Group C (Medium Pink/Scale 3), Group D (Pinkish Red/Scale 4), and Group E (Full Red/Scale 5). To establish a comprehensive data matrix for the hybrid classification model, a standardized suite of ten distinct pomological, physical, and chemical features was systematically evaluated and compared for each fruit sample across all groups and harvesting locations. The physical metrics comprised fruit weight, width, length, height, stem length, firmness (hardness), and kernel (seed) weight. Concurrently, the biochemical variables were determined through localized laboratory analysis, which included SSC, maturity levels via NaOH titration, and total titratable acidity. These extracted features, analyzed according to each specific sampling location alongside the synchronized field counts of adult cherry fruit flies, constituted the definitive input vector used in the downstream machine learning and rule-optimization processes.

### 2.3. Proposed Hybrid RF-ACO Model Architecture

This study presents a unique hybrid approach combining ensemble learning and nature-inspired meta-heuristic optimization techniques to optimize the inverse trade-off between high performance and interpretability, a common issue in machine learning models. The proposed model synthesizes the robust rule-generating capabilities of the RF algorithm with the global search and selection capabilities of the ACO algorithm in a single structure.

The methodological process essentially consists of the following stages:

Data Preprocessing and Boundary Setting: At the beginning of the process, the variance ranges of each feature in the raw dataset are analyzed to determine dynamic lower and upper bounds. This step ensures that the generated rules are confined to mathematically meaningful ranges and protects the model from overfitting. The data is then subjected to Min-Max normalization and label encoding to prepare it for the optimization phase.

RF-Based Candidate Rule Mining: The RF community, which acts as the rule generation engine for the model, is composed of N decision trees (estimators). Each tree is trained on different subsets of the data to ensure diversity. After training is complete, all branches of the trees, from the root node to the leaf node, are converted into logical classification paths. These paths are then converted into an “Interval-based” format, creating a massive pool of candidate rules. This approach incorporates not only the limited perspective of a single tree but also the collective knowledge of the entire forest into the system.

Optimal Rule Selection and Penalty Mechanism with ACO: In the second stage, the ACO algorithm is employed to select the most effective and compact subset from thousands of candidate rules. Artificial ants navigate the rule pool, creating rule combinations based on pheromone trails and intuitive information. The fitness function used here represents the model’s specificity: The function not only focuses on maximizing overall accuracy but also penalizes the “False Positive” (FP) rate with a specific weight. This penalty mechanism maximizes the model’s reliability, especially in areas where misclassification is costly (trap detection, cybersecurity, etc.).

Explainable Artificial Intelligence (XAI) and Output Analysis: The process ultimately results in a transparent structure that directly appeals to human logic, unlike complex and difficult-to-understand black-box models. By limiting the number of selected rules (e.g., 8–20 rules), the model’s complexity is reduced while maintaining high prediction accuracy. The resulting “IF-THEN” rules allow experts to trace step-by-step why the system made a particular decision.

**Figure 3:**
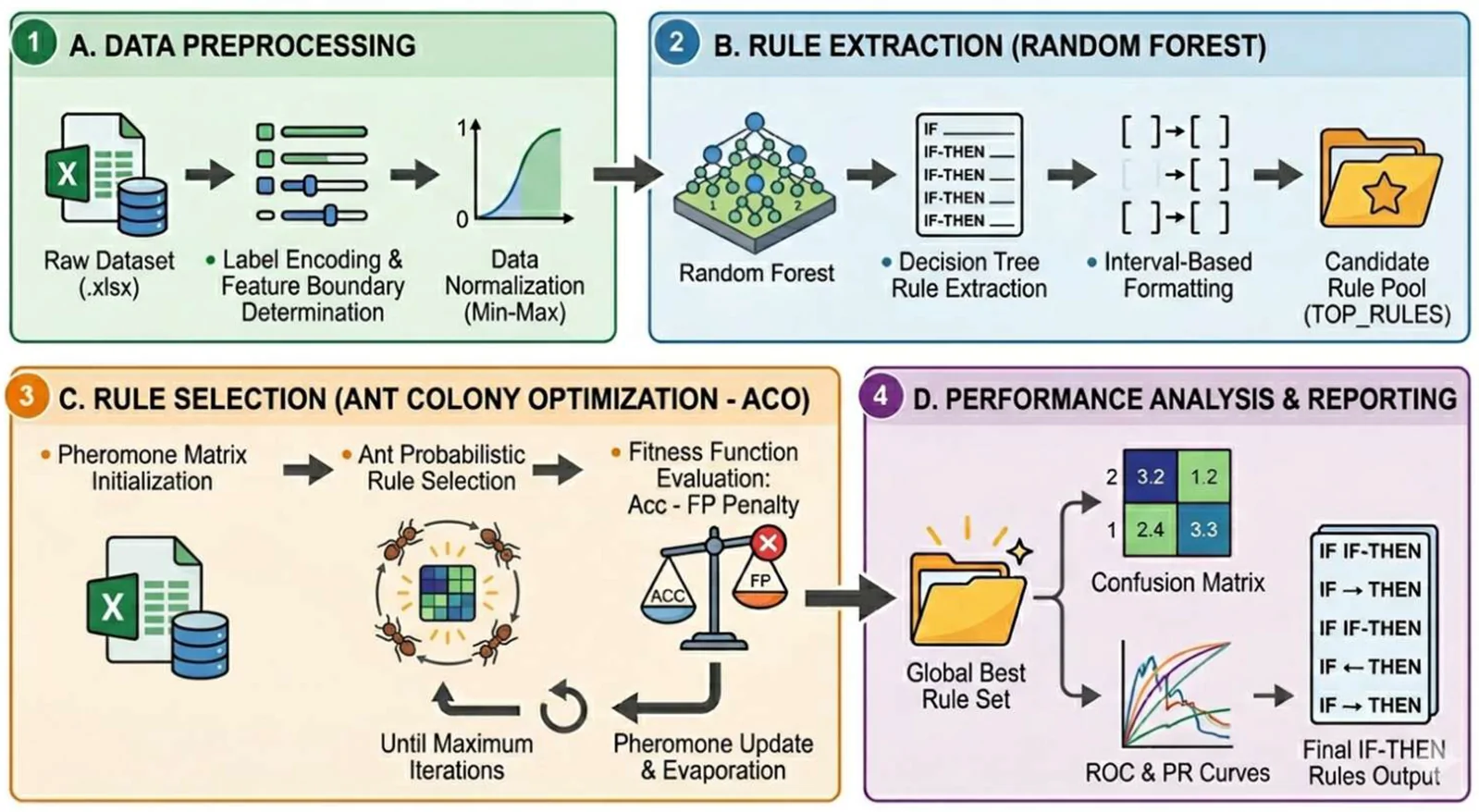
Proposed method.

The end-to-end architectural flow, components, and data traffic of the proposed method, from data entry to performance reporting, are detailed in Figure 3.

The experimental parameters used in the proposed RF-ACO model are summarized in Table 1. These settings were carefully chosen to balance classification accuracy, model reliability, and interpretability.

## 3. Results

**Figure 4:**
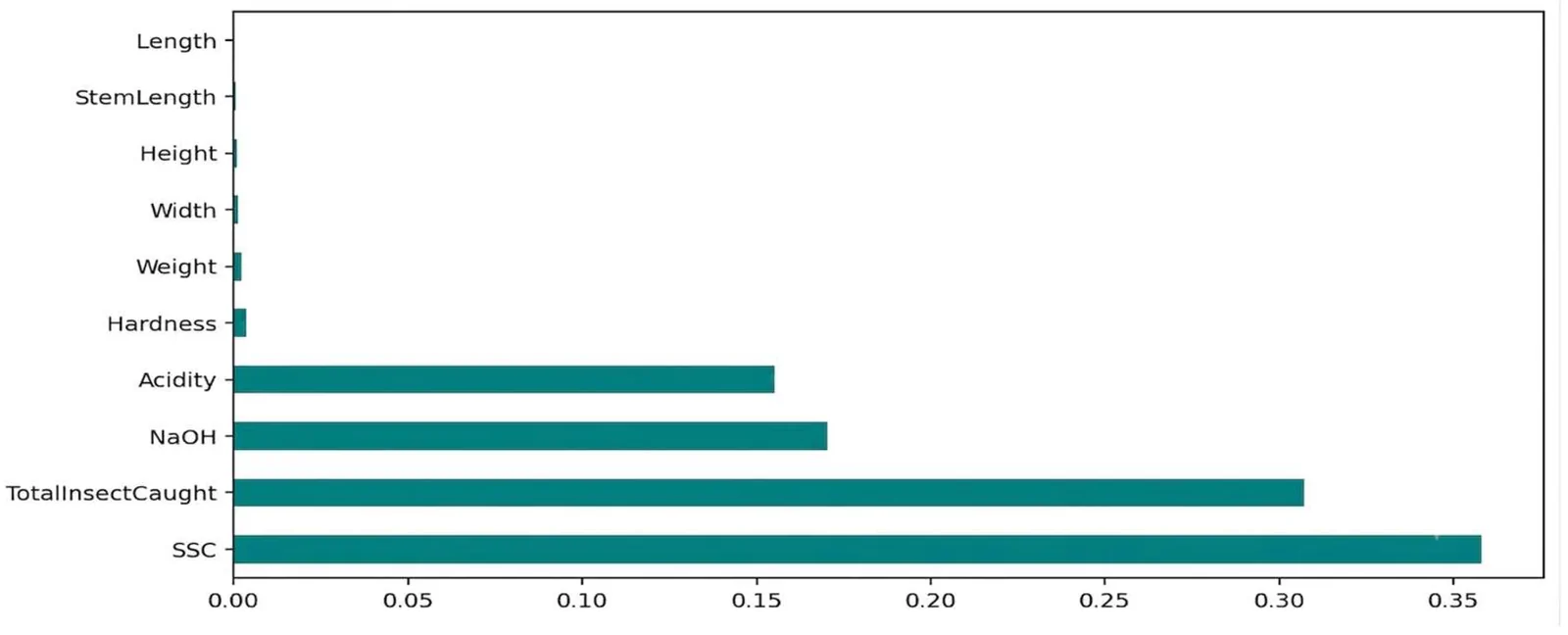
Ranking the attributes used in the decision-making process of the hybrid model according to their importance levels.

The results of the attribute significance analysis, conducted to determine which variables are more effective in the model’s decision-making process, are presented in Figure 4. Examining the graph clearly shows that chemical and biological parameters are the variables with the most decisive role in classification success. According to Figure 4, the SSC variable stands out as the model’s strongest distinguishing feature with an importance score of approximately 36%. Following this, the TotalInsectCaught variable plays a dominant role in decisions with an impact factor of over 30%. The levels of NaOH and Acidity, which are chemical parameters, also have significant weight in the model’s classification logic. On the other hand, it is observed that morphological features (Length, Stem Length, Height, etc.) and physical attributes (Weight, Hardness), as presented in Figure 4, have a much lower importance in determining target classes compared to chemical data. This hierarchical structure is based on RF-based rule generation, which scientifically explains why the qualified rules generated during the rule-making process are predominantly based on these four fundamental variables listed above.

**Figure 5:**
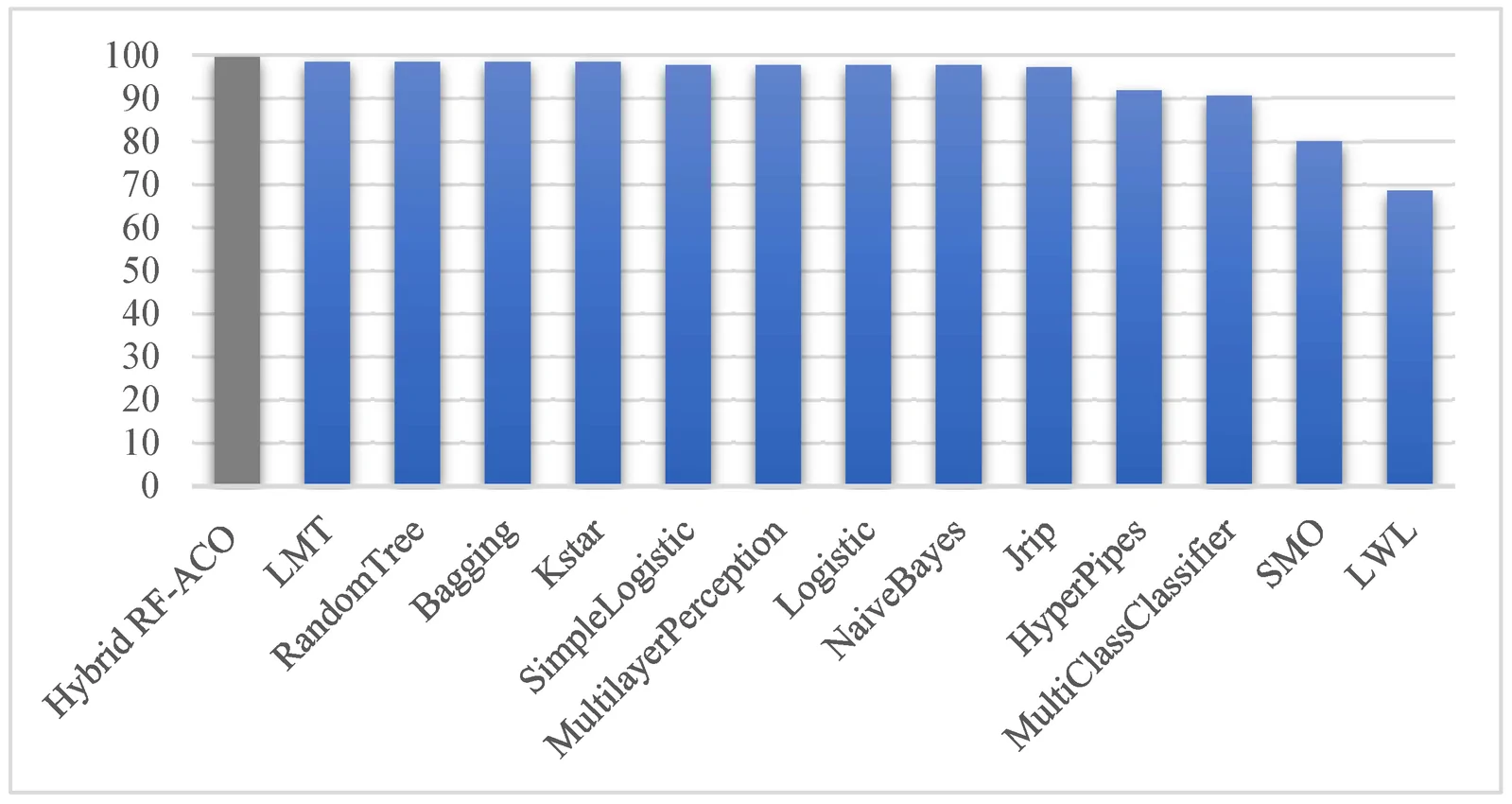
Accuracy-based comparison of the proposed model with other classification algorithms.

Figure 5 compares the overall classification accuracy of the proposed Hybrid RF-ACO model with 14 different machine learning algorithms frequently preferred in the literature. A detailed examination of the graph reveals that the developed Hybrid RF-ACO model achieves a remarkably high accuracy rate of 99.48%, surpassing all its competitors and ranking first. This proposed hybrid approach is compared with LMT and Random Forest Machine Learning algorithms, each achieving an accuracy rate of 98.42%. Tree, Bagging, and Kstar are powerful tree/community-based algorithms that are followed; this validates the success of tree architectures in analyzing non-linear agricultural data structures such as cherry pomology and pest density, while the integration of ACO proves that it further enhances the current success. Models in the middle of the list, such as SimpleLogistic, MultilayerPerception, Logistic, and NaiveBayes, exhibit a stable performance of 97.64%, while the rule-based Jrip algorithm shows a performance of 97.11%. However, they lag behind the proposed model in terms of transparency of decision mechanisms and “IF-THEN” rule generation capability. Models at the bottom of the comparison, HyperPipes (91.83%) and MultiClassClassifier (90.55%), show a need for optimization. In particular, the SMO (79.99%) and LWL (68.50%) algorithms proved significantly inadequate in their ability to generalize to this agro-ecological dataset, which contains multi-class variations across five different coloration periods. As a result, Figure 3, RF ACO-based architecture, which filters and optimizes the raw rules it produces using a meta-heuristic approach, clearly demonstrates its methodological superiority over many traditional and modern single/community learning models.

**Figure 6:**
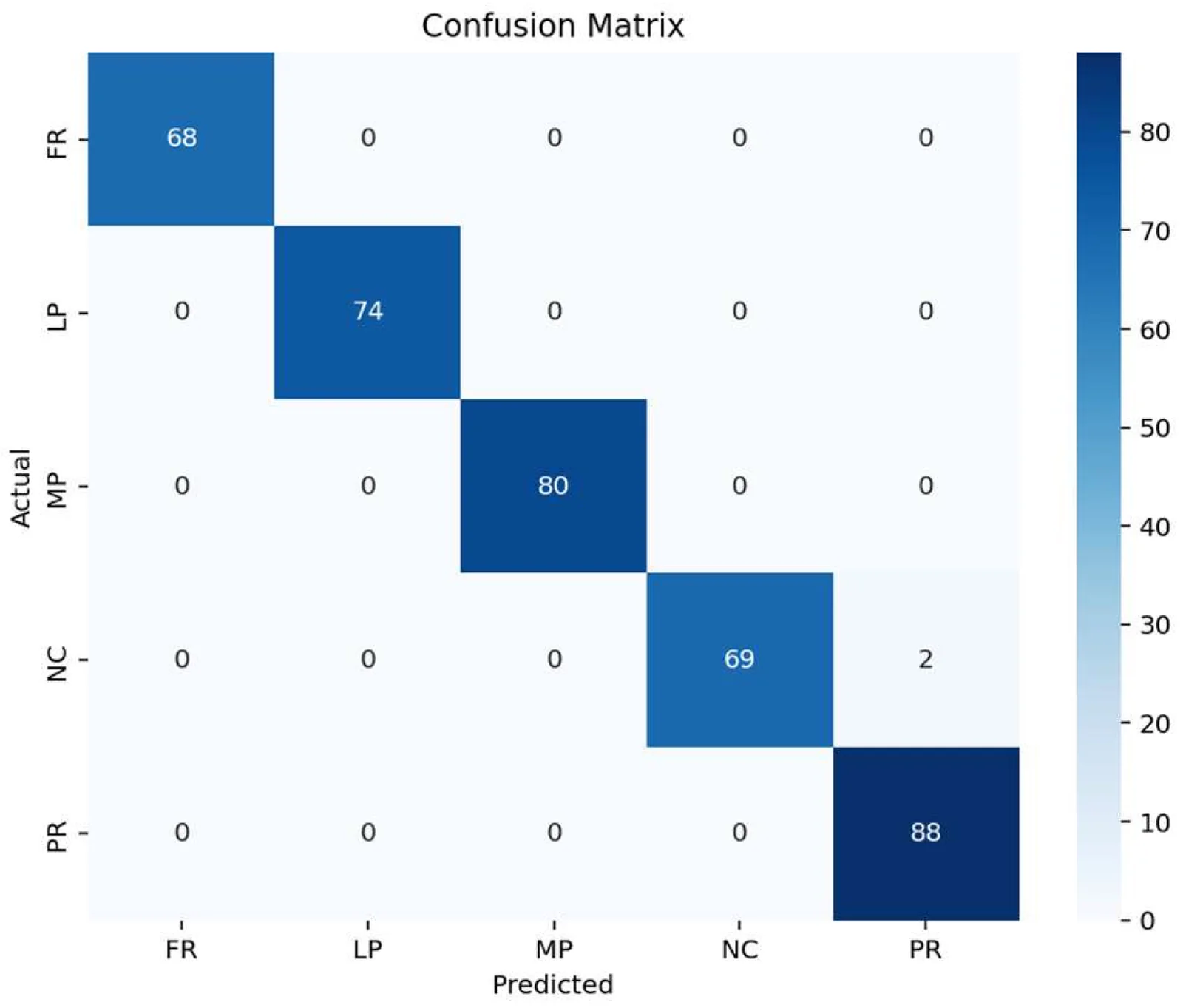
Confusion matrix illustrating the classification performance of the proposed Hybrid RF-ACO model.

The proposed hybrid RF-ACO model is detailed in the complexity matrix presented in Figure 6. The matrix clearly reveals the model’s accuracy across five different categories: FR, LP, MP, NC, and PR. Analysis of the data in Figure 4 shows that the model achieved a high overall accuracy rate of 99.46%, with only 2 errors across a total of 373 test samples. Examining the model’s class-based performance yielded the following results:

- As shown in Figure 6, the model achieved a 100% accuracy rate in the FR (68/68), LP (74/74), MP (80/80), and PR (88/88) classes, with no mismatches occurring in these classes;
- The only error margin in the model was observed in the NC class. Figure 6 shows that 69 out of 71 NC samples were correctly predicted, while only 2 samples were confused with the PR class.

This high success rate demonstrates the effectiveness of the FP (False Positive) penalty mechanism with a coefficient of 4.0, integrated into the fitness function used in the ACO process. The results in Figure 6 confirm that the proposed method selects a reliable and highly discriminatory rule set by minimizing critical errors.

**Figure 7:**
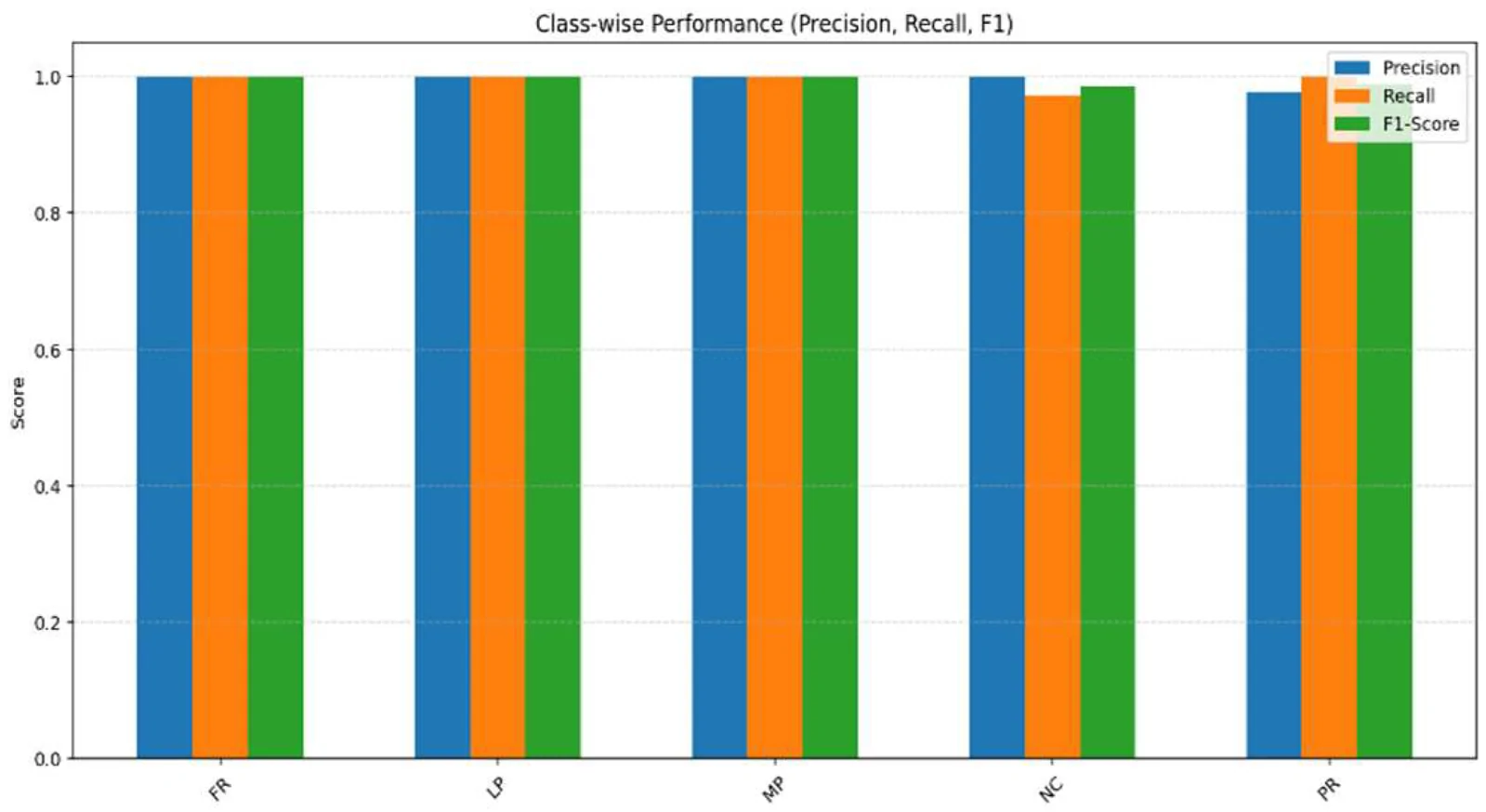
Class-wise performance comparison of precision, recall, and F1-score metrics on the test dataset.

Precision, recall, and F1-score values calculated to measure the success of the proposed hybrid RF-ACO model in each class are comprehensively presented in Figure 7. A general overview of the graph reveals that the model exhibits exceptional stability across all performance metrics, ranging from 0.97 to 1.00. The fact that all three metrics scored perfectly for the FR, LP, and MP classes confirms that these classes are distinguished without error, as seen in Figure 7, and that the rule set operates with maximum selectivity on these categories. While the very small decrease in sensitivity observed for the NC class reflects a limited number of misclassifications in the complexity matrix, the class’s F1-score remains quite high, preventing any disruption to the overall stability of the model. In the PR class, although the precision value shows a slight deviation, the perfect score for sensitivity indicates that the model captures all samples belonging to this class, but occasionally makes erroneous transfers from other classes.

**Figure 8:**
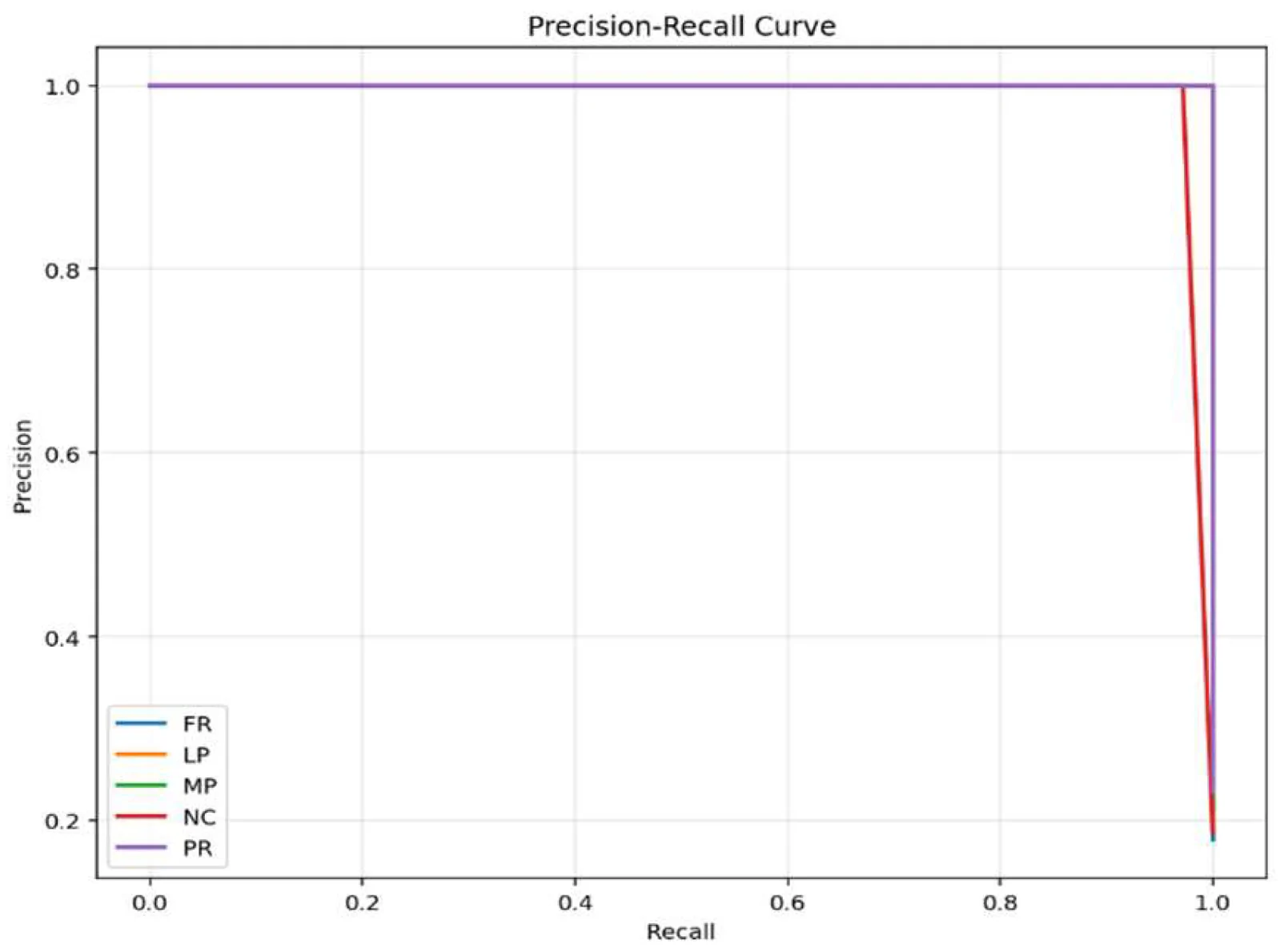
Class-based Precision Recall curves and performance consistency of the Proposed RF-ACO model.

The Precision Recall curves, created to verify the stability of the classification performance and the class-distinguishing power of the proposed hybrid model, are presented in Figure 8. This graph is critically important, especially for understanding the class distributions in the dataset and how confidently the model predicts samples belonging to each class. Examining Figure 8, it is clearly seen that the curves for all classes, FR, LP, MP, NC, and PR, are clustered in the upper right corner of the graph (at coordinates 1.0, 1.0) and cover a wide area. This scientifically proves that the model can maintain a very high precision rate without sacrificing recall. The curves following an ideal right angle indicate that the rule set optimized with the ACO algorithm not only makes correct predictions but also exhibits high prediction consistency by minimizing the false positive rate for all classes.

**Figure 9:**
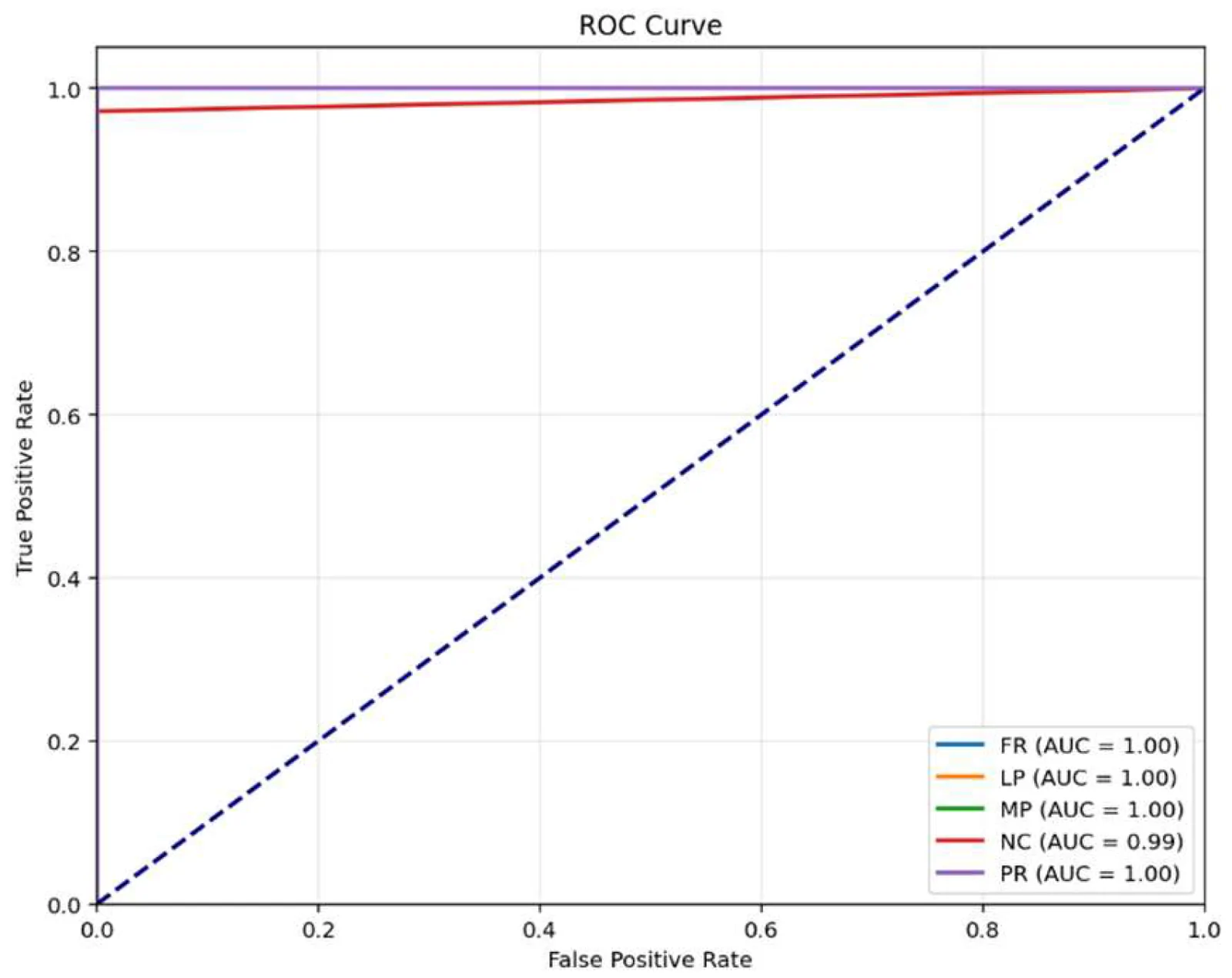
ROC curves and AUC (Area Under the Curve) performance values for the classes on the test dataset.

The Receiver Operating Characteristic (ROC) curves, created to validate the class discrimination ability and overall prediction performance of the proposed hybrid RF-ACO model, are presented in Figure 9. Examination of the graph shows that the area under the curve (AUC) reaches a perfect level of 1.00 for all four classes: FR, LP, MP, and PR. This demonstrates that the model can discriminate between these four classes without error, maximizing the true positive rate (TPR) while keeping the false positive rate (FPR) close to zero. The AUC value of 0.99 obtained for the NC class in Figure 9 confirms that the model also exhibits extremely high discrimination in this class, offering stable performance with only a very small margin of error. The fact that all class curves are perpendicular to the upper left corner indicates that the model is moving away from the random prediction line (diagonal dashed line) to the maximum extent, approaching ideal classifier performance.

**Figure 10:**
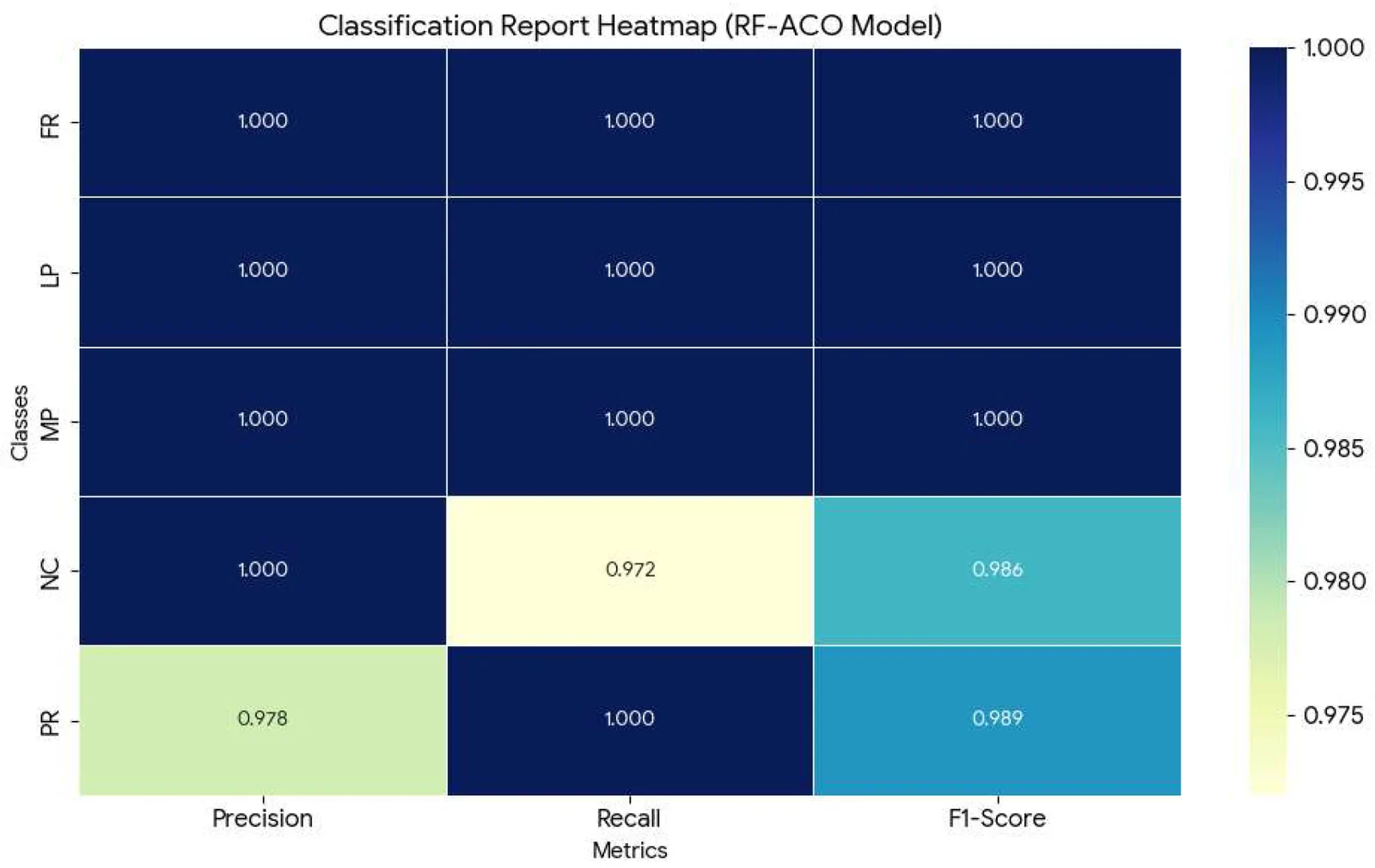
Heatmap class-based analysis performance metrics (Accuracy, Sensitivity, F1-score) of the Hybrid RF-ACO model.

The proposed hybrid model, to more clearly analyze the distribution of success among the classes, is presented in Figure 10. This graph shows the distribution of precision, recall, and F1-score values according to classes using a color scale. Examining Figure 10, it can be seen that the FR, LP, and MP classes are represented by dark color tones, achieving a perfect score of 1.000 in all metrics and exhibiting flawless separation. The sensitivity value for the NC class remaining at 0.972 is due to some samples from this class being confused with the PR class, while the slight deviation of 0.978 in the precision value of the PR class is due to the model labeling some samples from the NC class as PR. However, the fact that the F1-scores of all classes are 0.986 and above visually proves in Figure 8 as a whole that the model has an extremely balanced and highly reliable classification capacity.

The proposed hybrid RF-ACO model and sample rules selected from the total rule set generated by the system are presented in Table 2. This table was prepared to represent how the model makes clear and traceable inferences from a complex dataset.

Table 2 reveals the following key conclusions:

- Representative Sample: The rules presented in the table represent only a portion of the total rule pool, but reflect the characteristic limits set by the model for each class (PR, NC, LP, MP).
- Rule Quality: The fact that all the sample rules have an accuracy value of 1.0 (100%) proves that the ACO algorithm creates a refined structure by filtering only the rules with the highest discrimination among thousands of candidate rules.
- Variable Interaction: The logical expressions in Table 2 clearly show at what threshold values parameters such as SSC, NaOH, Acidity, and Hardness combine to produce definitive classification decisions.

In conclusion, Table 2 provides qualified evidence that, unlike black box models, the decision-making process of the proposed system is auditable, transparent, and interpretable by experts.

## 4. Discussion

In this study, five different coloration stages of cherry fruit were classified using a hybrid RF-ACO model developed with data on pomological characteristics and cherry fly density. The results showed that the proposed method exhibited a highly successful performance with an accuracy rate of 99.48%. Furthermore, thanks to the explainable IF-THEN rules generated by the model, not only was high classification success achieved, but the decision-making mechanism was also interpretable by experts.

In the feature significance analysis, the identification of SSC, total number of cherry flies captured, NaOH, and acidity values as the most important variables revealed a strong relationship between fruit ripening and pest density. Specifically, the identification of the total number of cherry flies as the second most important variable indicates that the pest population is directly related to the fruit coloration stages.

The results obtained support the findings of the study conducted by Akyol et al. [14]. In that study, the aim was to distinguish the second coloration period, where the cherry fly is most prevalent, from other periods, and it was stated that this period constitutes a critical threshold for the pest. The RF-ACO model developed in this study was able to distinguish not only the second coloration period but all five different coloration periods with high accuracy within the same model. This shows that the proposed approach offers a more comprehensive decision support structure compared to existing studies.

The research results reveal that the second coloration stage (the less pink stage) is a critical biological phase for the cherry fly. The color change, sugar accumulation, and changes in chemical composition occurring in the fruit during this period stimulate the pest’s egg-laying behavior. Indeed, the literature also reports that the cherry fly prefers fruits that are not fully mature but have begun to color for egg laying. Therefore, the second coloration stage is considered one of the periods with the highest risk of economic damage from the cherry fly. The prominence of maturity indicators such as cherry fly density, SSC, and acidity in the rules generated by the model confirms the pest’s sensitivity to changes in fruit physiology.

The physiological thresholds embedded in the extracted IF-THEN rules exhibit strong consistency with the known ripening biology of sweet cherry (Ziraat 900). Specifically, key decision attributes such as SSC, firmness, and titratable acidity directly reflect fruit maturation dynamics. During the transition to the second coloration stage (Scale 2 to Scale 3), the fruit undergoes rapid sugar accumulation (SSC ranges of approximately 13.9–15.6%) accompanied by a steady decline in organic acids. This specific physiological window acts as a critical biological trigger for *Rhagoletis cerasi*, as ovipositing females preferentially target fruits entering the initial softening and sweetening phases. Because the experimental dataset was harvested across four distinct commercial orchards (Harput 1, Harput 2, Baskil 1, Baskil 2) encompassing variations in elevation (980–1210 m a.s.l.) and microclimate, the strong performance underscores the model’s robustness across different field locations. However, as these biochemical thresholds are inherently cultivar-specific to Ziraat 900, future studies evaluating multi-year sampling (temporal extrapolation) and testing across diverse cherry cultivars will further confirm the broad scalability of the proposed framework.

The study demonstrates that optimizing the extensive rule pool produced by Random Forest with the ACO algorithm significantly enhances discrimination between phenological classes. Furthermore, the implementation of a custom fitness function that explicitly penalizes false positive errors has contributed to obtaining highly reliable classification results, which is essential for agricultural decision support where pest control interventions are planned.

From an agricultural perspective, the developed RF-ACO model offers significant decision-support advantages to growers and technical personnel. Using objective pomological measurements alongside trap counts, the model enables highly accurate determination of the fruit’s coloration stage. This facilitates more precise planning of targeted cherry fly spraying times. Specifically, accurately predicting the second coloration stage—where the pest causes severe economic damage—will contribute to reducing unnecessary broad-spectrum pesticide applications, lowering production costs, and minimizing environmental impact.

In conclusion, the proposed hybrid RF-ACO model is an effective method that can classify the coloration stages of cherry fruit with high accuracy, generate explainable decision rules, and offer applicable decision support mechanisms in cherry fly control. Future integration of the model with mobile applications, smart trap systems, and precision agriculture technologies will provide significant opportunities for sustainable pest management in cherry production.

In this study, fruit sampling and adult fly monitoring were conducted synchronously across five distinct phenological coloration stages (Scale 1 to Scale 5). It is important to clarify that pomological and biochemical parameters (e.g., firmness, titratable acidity, SSC) alongside trap-based adult fly counts served as the primary independent predictor variables (features), whereas the 5 phenological coloration stages (Scale 1 to Scale 5: NC, LP, MP, PR, FR) served as the classification target class. This synchronous cross-sectional sampling design was deliberately adopted to capture an immediate physiological snapshot of the host-pest interaction at specific fruit maturity milestones. By identifying how physical, biochemical, and pest population attributes jointly define host maturation, the model provides growers with actionable insights to objectively determine the exact phenological stage without relying solely on subjective visual checks. However, we acknowledge the inherent limitations of synchronous cross-sectional data, which reflect concurrent physiological relationships rather than prospective early-warning forecasts. To further enhance the predictive forecasting capabilities and real-time early warning applicability of the model, future studies will incorporate temporally lagged variables (e.g., utilizing trap capture data from previous weeks, t-1, to predict maturity and infestation risk at stage t) and perform sensitivity analyses.

## 5. Conclusions

In this study, a unique hybrid RF-ACO model was developed for the simultaneous and multi-class classification of five different coloration stages (Scales 1–5) in cherry fruit ripening processes, using pomological characteristics and cherry fly density data. The proposed model eliminates the “black box” structure, one of the biggest disadvantages of traditional machine learning approaches, and successfully offers both high classification accuracy and a transparent decision mechanism that can be interpreted by experts. The experimental results showed that the hybrid RF-ACO model achieved a very high accuracy rate of 99.48%, surpassing 14 other machine learning algorithms commonly used in the literature. According to the feature significance analysis results, SSC with an impact factor of 36% and the total number of insects captured (cherry fly density) with an impact factor of over 30% stood out as the most decisive variables in the classification process. While chemical parameters such as NaOH and acidity levels were considered, it was determined that morphological and physical characteristics had a lower level of discriminatory power. The highly accurate “IF-THEN” rules generated by the model were mathematically and logically validated at what threshold values these variables combine to form the target classes.

In future studies, the proposed hybrid RF-ACO framework will be extended by inverting the predictive architecture. Specifically, easily obtainable, non-destructive visual and physical features (e.g., coloration stages and fruit dimensions) will be utilized as input variables to predict harder-to-measure internal biochemical attributes and real-time *Rhagoletis cerasi* infestation risks. This inverted approach aims to facilitate the integration of our model into IoT-based field sensor networks and autonomous smart IPM systems.

## Figures and Tables

**Table 1:** Experimental Parameter Settings for the Proposed RF-ACO Model.

Stage	Parameter	Value	Description
RF	N_Estimators	80	Number of decision trees trained to generate the rule pool.
	Max_Depth	8	The maximum depth allowed for each tree.
	Min_Samples_Leaf	2	Minimum number of samples required at a leaf node.
	Bootstrap	True	Method used for sampling diversity during tree training.
Rule Pool	Top_Rules	500	Number of the highest accuracy rules selected for optimization.
ACO	Population Size	40	Number of artificial ants per iteration.
	Max_Iteration	120	Maximum iteration count
	Min_Rules	8	Minimum number of rules required in the final model.
	Margules	20	Maximum rules allowed to maintain interpretability.
Fitness Function	FP_Penalty_Weight	4	Weight coefficient used to penalize false positive errors.
	Global Accuracy	Metric	The primary objective for evaluating the selected rule set.

**Table 2:** High-accuracy sample decision rules selected from the total rule set generated by the Proposed RF-ACO hybrid model.

Rule	Class	Accuracy
IF 13.900 ≤ SSC ≤ 15.600 AND 0.600 ≤ Hardness ≤ 2.250 AND 8.830 ≤ NaOH ≤ 11.795 AND 13.010 ≤ Height ≤ 18.015	PR	1.00
IF 2.700 ≤ Hardness ≤ 10.000 AND 10.195 ≤ NaOH ≤ 15.480 AND 13.010 ≤ Height ≤ 16.370	NC	1.00
IF 8.830 ≤ NaOH ≤ 13.750 AND 0.000 ≤ TotalInsectCaught ≤ 2.500 AND 16.370 ≤ Height ≤ 19.810 AND 15.230 ≤ Length ≤ 19.775 AND 2.250 ≤ Hardness ≤ 10.000 AND 0.555 ≤ CoreWeight ≤ 0.740	NC	1.00
IF 8.830 ≤ NaOH ≤ 13.750 AND 0.000 ≤ TotalInsectCaught ≤ 2.500 AND 16.370 ≤ Height ≤ 19.810 AND 19.775 ≤ Length ≤ 29.890 AND 2.250 ≤ Hardness ≤ 10.000 AND 0.591 ≤ Acidity ≤ 0.662	LP	1.00
IF 19.410 ≤ Length ≤ 29.890 AND 8.830 ≤ NaOH ≤ 9.785 AND 0.615 ≤ Acidity ≤ 1.067	MP	1.00
IF 12.700 ≤ SSC ≤ 12.950 AND 20.195 ≤ Width ≤ 23.260	LP	1.00
IF 15.230 ≤ Length ≤ 18.660 AND 0.615 ≤ Acidity ≤ 0.655 AND 0.500 ≤ TotalInsectCaught ≤ 27.000	MP	1.00
IF 14.440 ≤ StemLength ≤ 50.365 AND 10.045 ≤ NaOH ≤ 13.225 AND 0.733 ≤ Acidity ≤ 1.067 AND 0.000 ≤ TotalInsectCaught ≤ 0.500 THEN	NC	1.00
IF 12.700 ≤ SSC ≤ 15.600 AND 11.795 ≤ NaOH ≤ 15.480 AND 0.500 ≤ TotalInsectCaught ≤ 10.000 AND 0.600 ≤ Hardness ≤ 2.350 AND 0.655 ≤ Acidity ≤ 1.067	MP	1.00
IF 0.591 ≤ Acidity ≤ 0.615	LP	1.00

## Data Availability

The data presented in this study are available on request from the corresponding author. The data are not publicly available due to project and institutional restrictions.

## Abbreviations

The following abbreviations are used in this manuscript:

ACO	Ant Colony Optimization
AW-IPM	Area-Wide Integrated Pest Management
FR	Full Red (Scale 5)
IPM	Integrated Pest Management
LP	Light Pink (Scale 2)
MP	Medium Pink (Scale 3)
NC	No Color (Scale 1)
PR	Pinkish Red (Scale 4)
RF	Random Forest
SSC	Soluble Solids Content
XAI	Explainable Artificial Intelligence

## References

[1] Kaplan M. (2019). Elazığ İli Kiraz Üretim Alanlarında Kiraz Sineği (*Rhagoletis cerasi* L.) (Diptera:Tephritidae)’nin Yayılışı, Doğaya Çıkış Zamanı, Popülasyon Gelişimi Ve Bulaşıklık Oranını Belirlenmesi. Eur. J. Sci. Technol..

[2] Fimiani P., Cavalloro R. (1983). Multilarval infestations by *Rhagoletis cerasi* L. (Diptera: Tephritidae) in cherry fruits. Fruit Flies of Economic Importance.

[3] Birişik N., Birişik N. (2018). Teoriden Pratiğe Kimyasal Mücadele Ve Gelecek Stratejisi. Teoriden Pratiğe Kimyasal Mücadele.

[4] Ahamed K.U., Islam M., Uddin A., Akhter A., Paul B.K., Yousuf M.A., Uddin S., Quinn J.M.W., Moni M.A. (2021). A deep learning approach using effective preprocessing techniques to detect COVID-19 from chest CT-scan and X-ray images. Comput. Biol. Med..

[5] Özgen İ., Güral Ö.Ü.Y. (2024). The effect of insecticides used against the cherry fly *Rhagoletis cerasi* L. (Diptera: Tephritidae) at different cherry fruit colouring periods on fruit worm rates. J. Entomol. Zool. Stud..

[6] Rattanapun W., Amornsak W., Clarke A.R. (2009). *Bactrocera dorsalis* preference for and performance on two mango varieties at three stages of ripeness. Entomol. Exp. Appl..

[7] Wijekoon W.M.C.D., Ganehiarachchi G.A.S.M., Wegiriya H.C.E., Vidanage S.P. (2024). Seasonal forecasting of *Bactrocera dorsalis* Hendel, 1912 (Diptera: Tephritidae) in bioclimatic zones of Sri Lanka using the SARIMA model. CABI Agric. Biosci..

[8] van Ginkel Bekker G.F.H., Addison M., Addison P., van Niekerk A. (2019). Using machine learning to identify the geographical drivers of *Ceratitis capitata* trap catch in an agricultural landscape. Comput. Electron. Agric..

[9] Alencar Filho E.B., Guimarães R.P., Santos V.C., Bispo B.A.G., Paranhos B.A.G., Aquino N.C., Nascimento R., Oliveira Neto R.F. (2025). Converging XGboost Machine Learning and Molecular Docking Strategies to Identify Attractants for *Ceratitis capitata*: Molecular Characterization and Database Curation of Natural Ligands for In Vitro/In Vivo Tests. Arch. Insect Biochem. Physiol..

[10] Córdova-García G., Tapia-Mcclung H., Rao D., Pérez-Staples D. (2025). Inferring tephritid fly pupal development stage using non-invasive near infrared imaging and machine learning classification. Bull. Entomol. Res..

[11] Leonardo M.M., Carvalho T.J., Rezende E., Zucchi R., Faria F.A. Deep Feature-Based Classifiers for Fruit Fly Identification (Diptera: Tephritidae). Proceedings of the 2018 31st SIBGRAPI Conference on Graphics, Patterns and Images (SIBGRAPI).

[12] Nazir N., Fatima S., Aasim M., Yaqoob F., Mahmood K., Ali S.A., Awan S.I., ul Haq I. (2024). *Zeugodacus* fruit flies (Diptera: Tephritidae) host preference analysis by machine learning-based approaches. Comput. Electron. Agric..

[13] Akyol E., Alatas B., Ozgen I. (2025). Evolutionary Algorithm Approaches for Cherry Fruit Classification Based on Pomological Features. Agriculture.

[14] Yildirim S., Ozgen I., Alatas B., Yildirim H. (2026). Chaos-Embedded Multi-Objective Intelligent Optimization-Based Explainable Classification Model for Determining Cherry Fruit Fly Infestation Levels Using Pomological Data. Biomimetics.

